# Reverse transcriptional profiling: non-correspondence of transcript level variation and proximal promoter polymorphism

**DOI:** 10.1186/1471-2164-6-110

**Published:** 2005-08-17

**Authors:** Rebecca Petersen Brown, Martin E Feder

**Affiliations:** 1 Department of Organismal Biology & Anatomy, The University of Chicago, Chicago, IL 60637, USA

## Abstract

**Background:**

Variation in gene expression between two *Drosophila melanogaster *strains, as revealed by transcriptional profiling, seldom corresponded to variation in proximal promoter sequence for 34 genes analyzed. Two sets of protein-coding genes were selected from pre-existing microarray data: (1) those whose expression varied significantly and reproducibly between strains, and (2) those whose transcript levels did not vary. Only genes whose regulation of expression was uncharacterized were chosen. At least one kB of the proximal promoters of 15–19 genes in each set was sequenced and compared between strains (Oregon R and Russian 2b).

**Results:**

Of the many promoter polymorphisms, 89.6% were SNPs and 10.4% were indels, including homopolymer tracts, microsatellite repeats, and putative transposable element footprints. More than half of the SNPs were changes within a nucleotide class. Hypothetically, genes differing in expression between the two strains should have more proximal promoter polymorphisms than those whose expression is similar. The number, frequency, and type of polymorphism, however, were the same in both sets of genes. In fact, the promoters of six genes with significantly different mRNA expression were identical in sequence.

**Conclusion:**

For these genes, sequences external to the proximal promoter, such as enhancers or in *trans*, must play a greater role than the proximal promoter in transcriptomic variation between *D. melanogaster *strains.

## Background

Transcriptional profiling via whole genome arrays has revealed both impressive diversity and unexpected commonalities in gene expression. Some fraction of this variation results in the various functional, developmental, and reproductive phenotypes that are downstream of gene expression [1,2]. The source of this variation, however, is in the nucleotides that regulate gene expression (as reviewed in [3]). Given the relationship between these nucleotides and gene expression, it should in principle be possible to reverse this relationship and retrodict the nucleotide sequences that have given rise to variation in gene expression. Indeed, this is the basis for numerous bioinformatic efforts to identify novel *cis*-regulatory motifs if not to elucidate the entire *cis*-regulatory code [4,5] from sequence that is conserved among genes with similar expression patterns. Here we ask a somewhat different but equally fundamental question of sequence that varies among genes with dissimilar expression: Are regulatory regions different for genes differing in expression, and similar for genes not differing in expression?

We ask this question of *cis*-regulatory sequence immediately upstream of the transcription start site and of similarities or differences in gene expression between members of the same species. Candidate *cis*-regulatory variation includes polymorphisms in transcription factor binding sites, multiallelic tandem repeat variation, such as microsatellites, single nucleotide polymorphisms (SNPs), and transposable element insertions. These polymorphisms can alter transcription rates and affect mRNA expression levels *in vivo *[6-10]. Functional *cis*-regulatory variation is widespread in the human genome [11] and these sites have a high degree of variability (about 0.6% of these sites are polymorphic [12]). Clearly the source of variation in gene expression need not lie in the proximal promoter and could be either in *cis *but far up- or downstream, or in *trans*. Lack of correlated variation in proximal promoter sequence and gene expression can be viewed as implicating these alternative sources.

We focus on variation in gene expression among members of the same species because this is the raw material of evolution. For evolutionary change to ensue by ordinary Darwinian mechanisms, variation within a species must exist, be heritable, and be consequential for fitness. Both classical [13,14] and recent [15-17] and others reviewed in [18] works implicate variation in gene expression among members of a population, controlled by regulatory as opposed to coding sequence, as a principal source of such variation. Although simple sequence analysis of regulatory regions from diverse and related members of a species can readily ascertain whether sequence variation is present and heritable, ascertainment of impact on gene expression from sequence alone is less precise. Although differences in gene expression are not necessarily consequential for fitness, if variation in regulatory sequence affects gene expression (or that similarity in sequence reliably corresponds to no variation in gene expression), then intraspecific variability in regulatory sequence could be used as a preliminary screen for evolvability. Indeed, sequence analysis of regulatory regions may potentially assess the evolutionary forces that shape them [15].

To test this possibility, we mined the whole-genome transcriptional profiles of two near-isogenic strains (Oregon R and Russian 2b) of *Drosophila melanogaster*, here representing variation within species. The original data [1] are for genes whose expression does or does not vary between non-virgin adults of the Oregon R and Russian 2b strains. Of the 12017 genes examined, 527 do not differ between strains and 483 differ between strains regardless of sex. With no *a priori *knowledge of these genes' sequence variation or function, we have selected 15 and 19 representatives of each class, respectively. We chose genes whose regulation is yet uncharacterized to avoid bias due to over- or under-sampling of genes with known transcriptional regulation, and included both known transcription response elements/conserved motifs and uncharacterized sequence in our analysis. We now report whether or not their proximal promoter sequence varies and, if so, whether or not this variation corresponds to variation in gene expression.

## Results

Similar numbers of genes composed the two genesets. 483 candidate genes whose expression varied reproducibly and highly significantly between the strains with no sex or sex by line effects (*P *< 0.01 for line and *P *> 0.05 for sex and sex by line) composed the first geneset. The second geneset comprised 527 candidate genes whose expression did not vary at all (*P *> 0.1 for sex, line, and sex by line). The remaining 11007 genes, whose significance values fell in between these *P *values, were not analyzed. Within the first geneset, 172 genes were more actively transcribed in Russian 2b females than in Oregon R females and 311 genes in Oregon R than Russian 2b females. The expression of 154 genes was greater for Russian 2b males than for Oregon R males and 329 genes for Oregon R than Russian 2b males. Thus, expression was consistently greater for the Oregon strain than the Russian strain (Table 1).

**Table 1:** Categorization of microarray data set.

**Genes whose expression varies only between strains (*P *< 0.01) with no sex or sex by line effects (*P *> 0.05)**			**Genes whose expression does not vary between sex, strain, or sex by strain (*P *> 0.1)**
**Strain**	**Female**	**Male**	
	
Russian 2b	172	154	527
Oregon R	311	329	
**Total**	483	483	

Many genes in the first geneset are false positives. The expected number of candidate genes in the first geneset, 325, is less than 483, the observed number resulting in a False Discovery Rate (FDR) of 67.4%. Thus, of the 483 genes chosen in the first gene set, 67.4% or ~325 genes are called as significantly different when they are not. In other words, the expression of ~158 of the 483 genes in the first geneset presumably differs significantly between the two strains.

**Figure 1:**
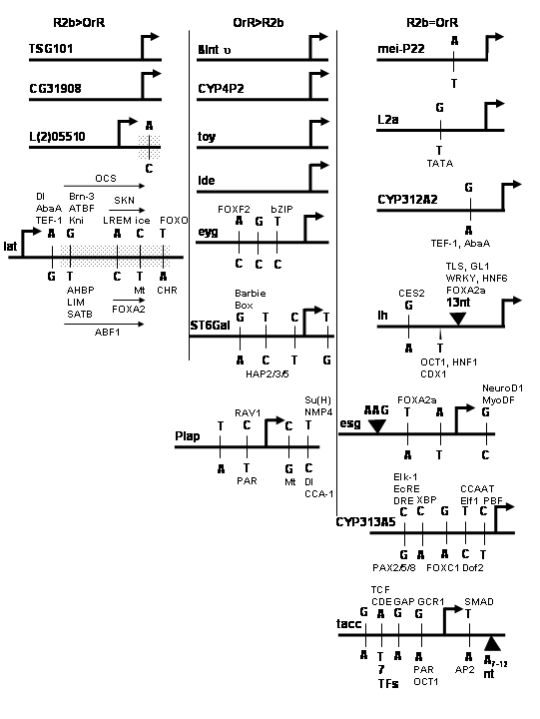
**Schematics of proximal promoters. **At least one kb of the proximal promoters of 34 candidate genes whose transcripts vary (left and center columns) or do not vary (right column) in expression between *D. melanogaster *strains. Genes whose expression is greater in the Russian 2b (R2b) strain are shown in the left column and those whose expression is greater in the Oregon R (OrR) strain in the center column. Genes with fewer polymorphisms are shown in Figure 1 and those with seven or more are shown in Figure 2. The long horizontal lines for each gene designate the sequence of the proximal promoter with changes in the R2b strain shown above the line and those in the OrR strain below the line. The large, bent arrows indicate transcriptional start sites. Single Nucleotide Polymorphisms (SNPs) are shown as small vertical lines and indels as triangles, with the sequence or length in nucleotides (nt). The numbers of SNPs are listed where there are too many to illustrate clearly. Putative transcription factor binding sites created or removed by the SNP or indel are shown above or below the polymorphisms, with short horizontal lines or arrows designating the included polymorphisms. Because there are too many to illustrate clearly, putative transcription factor binding sites are not shown for *CYP9C1 *(CG3616), *Fkbp13 *(CG9847), *qkr58E-3 *(CG3584), *bin *(CG18647), *tensin *(CG9379), *Cry *(CG16963), *KP78a *(CG6715), *Cng *(CG7779), *Fer2 *(CG5952), *Mt2 *(CG10692). Shaded boxes designate introns in the 5'untranslated regions (UTRs).

**Figure 2:**
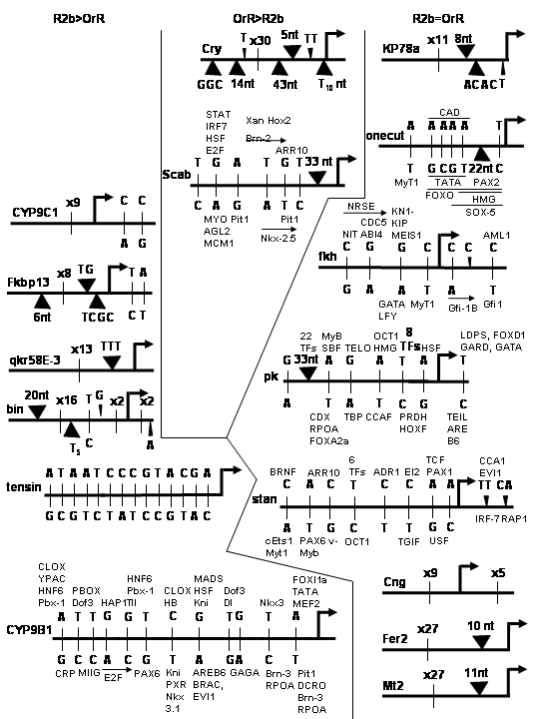
**Schematics of proximal promoters. **At least one kb of the proximal promoters of 34 candidate genes whose transcripts vary (left and center columns) or do not vary (right column) in expression between *D. melanogaster *strains. Genes whose expression is greater in the Russian 2b (R2b) strain are shown in the left column and those whose expression is greater in the Oregon R (OrR) strain in the center column. Genes with fewer polymorphisms are shown in Figure 1 and those with seven or more are shown in Figure 2. The long horizontal lines for each gene designate the sequence of the proximal promoter with changes in the R2b strain shown above the line and those in the OrR strain below the line. The large, bent arrows indicate transcriptional start sites. Single Nucleotide Polymorphisms (SNPs) are shown as small vertical lines and indels as triangles, with the sequence or length in nucleotides (nt). The numbers of SNPs are listed where there are too many to illustrate clearly. Putative transcription factor binding sites created or removed by the SNP or indel are shown above or below the polymorphisms, with short horizontal lines or arrows designating the included polymorphisms. Because there are too many to illustrate clearly, putative transcription factor binding sites are not shown for *CYP9C1 *(CG3616), *Fkbp13 *(CG9847), *qkr58E-3 *(CG3584), *bin *(CG18647), *tensin *(CG9379), *Cry *(CG16963), *KP78a *(CG6715), *Cng *(CG7779), *Fer2 *(CG5952), *Mt2 *(CG10692). Shaded boxes designate introns in the 5'untranslated regions (UTRs).

**Figure 3:**
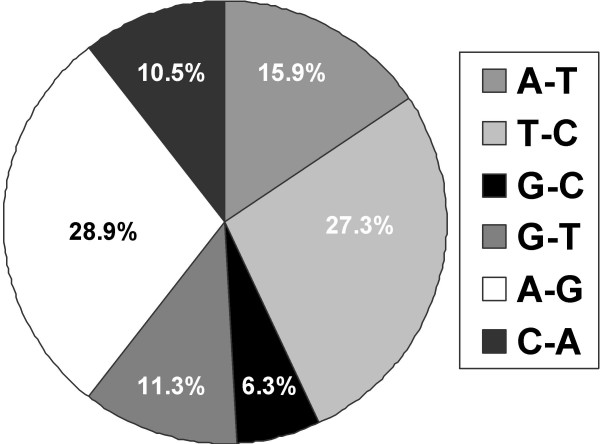
**Categories of SNPs between two strains. **We identified 258 single nucleotide polymorphisms (SNPs) in 1–2 kb of the promoter region 5' of the translational start site of genes whose expression does and does not vary on microarray. SNPs are reported here irrespective of the direction of change. Transitions, or changes within a nucleotide class such as A to G or C to T, comprise 56.2% of the number of SNPs, whereas transversions, or changes between nucleotide classes, occur less frequently.

Polymorphisms are numerous in both genesets. Of the 34 promoters analyzed, six do not differ between strains; however, they belong to genes whose transcripts differ between strains. The remaining 28 proximal promoters contain at least one polymorphism, with one promoter containing as many as 37 polymorphisms (Figures 1 and 2). The mean, median, and mode of polymorphisms per gene are 8.5, 6, and 0, respectively. Of the 288 total polymorphisms detected in at least 1 kb of the proximal promoters, 258 (89.6%) were SNPs and 30 (10.4%) were indels. Over half of the SNPs were transitions within a nucleotide class, while 43.8% were transversions between nucleotide classes (Figure 3). 239 putative binding sites for transcription factors were created or removed by these proximal promoter polymorphisms; thus 258/288 or 90% of proximal promoter polymorphisms fall within putative transcription factor binding sites (Figures 1 and 2).

Although few, indels varied between the strains by kind and from 1 to 43 nt in length [see Additional File 1]. Indels were classified as direct repeats (dr), homopolymer repeats (hpr), microsatellites (mcs), or non-repetitive (nr) according to designations of Schaeffer [19], page 165. In most cases, indels in one strain were the same as in the Celera strain used as a reference. However, six repeats in five different genes resulted in differences in sequence among the Russian 2b, Oregon R, and Celera strains. In *bin *(CG18647), five additional Ts are found in the Oregon R strain in comparison with the poly-T_4 _tract in the Russian 2b strain. This caused a transition and insertion from T_4_CT_3 _in the Celera strain. Distal to this in *bin*, the ATACCCGTACCCGTACCCAT sequence in the Russian 2b strain was shortened to ATACCCGTACCCAT in the Celera strain but absent altogether in the Oregon R strain. In *tacc *(CG9765), the poly-A tract varies from A_14–17 _nt among individuals in the Russian 2b strain, to A_23–26 _nt among individuals in the Oregon R strain, to A_22 _in the Celera strain. In *qkr58E-3 *(CG3584), a SNP and variation in the length of a poly-T tract resulted in T_3_GT_4 _in the Oregon R strain, T_9 _in the Celera strain and T_11 _in the Russian 2b strain. In *KP78a *(CG6715), the dinucleotide microsatellite AC was repeated 9X in the Russian 2b strain, 10X in the Celera strain, and 11X in the Oregon R strain. In *stan *(CG11895), the homopolymer repeat was T_10 _nt long in the Celera strain, T_11 _in the Russian 2b strain, and T_9 _in the Oregon R strain.

Indels in *Scab, Cry, Ih*, and *bin *(CG8095, CG16963, CG8585, CG18647) contained small regions (12–15 nt long) that shared sequence similarity with known transposable elements. Despite these matches, the sequences are not long enough to discriminate confidently between a TE footprint and chance occurrence of the same sequence. Flanking the indel in *Cry *listed in the Supplementary Table [see additional file 1], a 152 bp sequence from -889 to -1041 relative to the translational start site in the Oregon R strain matched a DNA LINE retroelement. In the same location in the Russian 2b strain a 152 bp sequence matched two overlapping *DNAREP1_DM *LINE elements [20].

The diversity and frequency of polymorphisms did not differ in proximal promoters from genes differing in expression and those with similar expression (*P *= 0.911 for indels, *P *= 0.935 for transition SNPs, and *P *= 0.842 for transversion SNPs) (Figures 1 and 2). For example, we identified 59 transversion SNPs, 76 transition SNPs (summing to 135 total SNPs), and 16 indels in the first geneset, and 54 transversion SNPs, 69 transition SNPs (123 total SNPs), and 14 indels in the second geneset. Also, the average promoter length in the first geneset was 1629 nt and 1620 nt in the second geneset (Table 2). Thus, for the 34 genes examined in this study, the lack of variation in proximal promoter sequence between the two genesets implicates alternative sources for divergent patterns of gene expression.

**Table 2:** Genes used in this study. From the data of Gibson *et al*. (2004), 34 candidate genes were chosen for study whose transcripts were expressed to higher levels in either Oregon R (OrR) or Russian 2b (R2b) strain (*P *< 0.01) (first geneset) or did not vary between strains (*P *> 0.1) (second geneset) and did not vary between the sexes or have sex by strain interaction (*P *> 0.05 or *P *> 0.1, for the two genesets respectively). (CODE, CG number from FlyBase Release 3.0; EXP. DIFF., indicates where gene expression on microarray does or does not vary between strains; N, number of replicate probes representing gene on microarray; CHR., chromosomal location; LEN., length of promoter analyzed in this study; ACC. #, GenBank accession numbers for gene's proximal promoter in the OrR strain followed by the R2b strain)

CODE	NAME	EXP. DIFF.	N	CHR.	FUNCTION	LEN.	ACC. #
CG1762	Bintv	OrR>R2b	4	2L	cell adhesion	1518	DQ017407,6
CG1944	CYP4P2	OrR>R2b	5	2R	steroid metabolism	1530	DQ017409,8
CG5105	Plap	OrR>R2b	5	2L	phospholipase A2 activator	1539	DQ017395,4
CG5952	Fer2	OrR = R2b	3	3R	transcription factor	1452	DQ017400,1
CG6547	mRpL2a	OrR = R2b	3	3R	ribosome structure	1448	DQ017398,9
CG7779	Cng	OrR = R2b	5	2R	cation channel	1622	DQ017397,6
CG8095	Scab	OrR>R2b	5	2R	cell adhesion	1652	DQ017403,2
CG9765	tacc	OrR = R2b	3	3R	microtubule binding	1428	DQ017405,4
CG10488	eyg	OrR>R2b	4	3L	transcription factor	1494	DQ017410,1
CG14827	mei-P22	OrR = R2b	4	3L	meiotic recombination	1392	DQ017412,3
CG1922	onecut	OrR = R2b	3	4	transcription factor	2410	DQ017414,5
CG3584	qkr58E-3	R2b>OrR	3	2R	RNA binding	1720	DQ017416,7
CG3616	CYP9C1	R2b>OrR	3	2R	unknown	1645	DQ017418,9
CG3758	esg	OrR = R2b	3	2L	transcription factor	1890	DQ017420,1
CG4485	CYP9B1	R2b>OrR	3	2R	unknown	1629	DQ017422,3
CG4871	ST6Gal	OrR>R2b	3	2R	polysaccharide metabolism	1776	DQ017424,5
CG5517	Ide	OrR>R2b	3	3L	unknown	1746	DQ017426,7
CG6715	KP78a	OrR = R2b	3	3R	protein kinase	1623	DQ017428,9
CG8585	Ih	OrR = R2b	3	2R	cation channel	1516	DQ017430,1
CG9847	Fkbp13	R2b>OrR	4	2R	protein folding	1676	DQ017432,3
CG10002	fkh	OrR = R2b	3	3R	transcription factor	1857	DQ017434,5
CG10094	CYP313A2	OrR = R2b	3	3R	steroid metabolism	1537	DQ017436,7
CG10692	Mt2	OrR = R2b	3	2L	DNA methylation	1577	DQ017438,9
CG11084	pk	OrR = R2b	3	2R	cell polarity	1523	DQ017440,1
CG11186	toy	OrR>R2b	3	4	transcription factor	2061	DQ017442,3
CG11895	stan	OrR = R2b	3	2R	GPC receptor	1636	DQ017444,5
CG15807	CYP313A5	OrR = R2b	3	3R	steroid metabolism	1390	DQ017446,7
CG16963	Cry	OrR>R2b	3	2L	eye lens structure	1769	DQ017448,9
CG18647	bin	R2b>OrR	3	3L	transcription factor	1595	DQ017450,1
CG4088	lat	R2b>OrR	3	2R	olfactory learning	2000	DQ017455,4
CG9379	by/tensin	R2b>OrR	3	3R	actin binding	1498	DQ017456,7
CG9712	TSG101	R2b>OrR	3	3L	ubiquitin-protein ligase	1374	DQ017453,2
CG13432	l(2)05510	R2b>OrR	3	2R	unknown	1404	DQ017458,9
CG31908	NA	R2b>OrR	2	2L	unknown	1328	DQ017460,1

## Discussion

The variation in proximal promoter sequence among the Oregon R, Russian 2b, and Celera strains, while extensive, typifies that among individuals and populations of eukaryotes. In a similar survey of 107 transcriptionally active genes in the human genome, Rockman and Wray [11] identified 140 experimentally validated *cis*-regulatory polymorphisms resulting in two-fold or greater variation in transcription rate and subsequent gene expression. In another survey of regulatory variation, Cowles *et al*. [21] found a relatively high frequency (6%) of *cis*-regulatory polymorphism (including SNPs, complex nucleotide repeats, insertions/deletions, and microsatellites) in the 1 kb region 5' of the predicted transcription start site of genes in four inbred mouse strains with allelic differences in expression by 1.5-fold or greater.

The frequencies of SNPs and indels were not independent. In fact, indels, including microsatellites, homopolymer repeats, and possibly TE footprints (see next paragraph), co-occurred with SNPs but SNPs could occur in the absence of indels. This suggests that indels occur infrequently and only after a promoter is poised for mutation via the presence of SNPs [22,23].

Some promoters have diverged remarkably between the two strains. For example, the *Cry *promoter contains 28 SNPs, 7 indels, and a 152 bp sequence including and flanking one indel that matches two different classes of transposable elements a DNA LINE element in the Oregon R strain and 2 overlapping *DNAREP1_DM *LINE elements in the Russian 2b strain [20]. This variation suggests that the *Cry *promoter is susceptible to mutation.

The polymorphisms are in regions within the proximal promoter that ought to affect gene expression. 90% of them fall within putative transcription factor binding sites (Figures 1 and 2). In addition, all regulatory information necessary for transcription in *Drosophila *is generally present within 1 kb of the basal promoter [24].

Surprisingly, genes differing in expression and genes not differing in expression had the same diversity and frequency of polymorphisms in their proximal promoters. Indeed, the proximal promoters from six genes whose expression differed were identical in sequence between the two strains. Because this region includes core regulatory sequences, polymorphisms between the two genesets (or their absence) ought reasonably be correlated with gene expression. In support of this expectation, 74% of transcription factor binding sites (as proxies for proximal promoter polymorphisms) in yeast genes are between 100 to 500 bases upstream of the translational start site and fewer than expected lie outside this region [25]. Thus, the non-concordance between proximal promoter sequence and gene expression is unexpected.

One possible explanation for this outcome is that the 34 genes examined are anomalous or unrepresentative of *Drosophila *genes in general. This explanation is unlikely. As discussed above, these genes' proximal promoters resemble other genes' in variation among individuals. Choice of these genes was also unbiased with respect to prior knowledge of transcriptional regulation, known transcriptional response elements, and mode of gene effect (e.g., dominance, underdominance, or additivity). Indeed, 5/34 (15%) of the blindly chosen genes were dominant, underdominant, or additive in their impact on gene expression, which compares favorably with the proportions of such effects for the *Drosophila *genome in general [1].

A second explanation for non-concordance between proximal promoter sequence and gene expression is that the expression of the genes in the first geneset actually does not differ between strains. To exclude this possibility, the first geneset included only genes whose expression profiles were different between strains (*P *< 0.01), and not between sexes or by the sex*strain interaction (*P *> 0.05 for both) [1]. These selection criteria confer a rather high False Discovery Rate of 67.4%. The actual statistical significance of variation between the two strains averages <0.0009 for the 19 genes in the first geneset. An additional 333 genes also meet this criterion. For these 352 genes (total), the FDR becomes ((0.0009)*(0.95)*(0.95)*(12017)*(3))/352 = 29.28/352 = 0.0832 or 8.3%. This low rate indicates 91.7% certainty that the 19 genes in this geneset truly differ in expression.

A third explanation is that some of these promoter variants may affect gene expression but that neutral sites dilute their regulatory activities. Functionally important regulatory sites may make up only a small fraction of the many promoter variants detected in the 1 kb region and the vast majority may be neutral. On the other hand, promoter variants whose expression levels are similar may be functionally equivalent (or neutral) as a result of stabilizing selection or epigenetic forces may even out promoter variation resulting in similar expression levels. Functional assays should be able to distinguish between these outcomes.

Another possibility is that the regulatory elements responsible for the observed variation in transcript abundance occur outside the proximal promoter, including in introns and 5' and 3' untranslated regions, and/or in *trans*. Several recent localizations of regulatory sequence also support this possibility. In a recent assignment of 142 expression phenotypes in humans to both *cis*- and *trans*-acting loci, 27 (19%) had a single *cis*-acting regulator (defined as regulators that map within 5 megabases of the target gene), 110 (77.5%) had a single *trans*-acting regulator (those that map elsewhere), and 5 (3.5%) had two or more regulators acting in *cis *and/or *trans *[26]. In budding yeast, *trans-*acting regulators were linked to 365 of 570 (64%) genes whose expression diverged between a laboratory and a wild strain of *Saccharomyces cerevisiae *whereas only 36% of divergent gene expression was linked to individual *cis*-acting loci [27]. Of 2294 genes whose expression differs between laboratory and wild yeast strains, 1716 (75%) map to 100–200 distinct *trans*-acting loci with widespread genomic effects and molecular functions but that do not encode transcription factors [28]. Of 3546 highly heritable transcripts in the same two strains of yeast, 3% map to a single locus, 17–18% to 1–2 loci, and half to more than 5 loci [29]. Using gene expression differences as quantitative traits, Schadt *et al*. [30] determined that genes involved in determining patterns of obesity in mice and humans map to QTLs other than the genes themselves. Many genes map to the same QTL, suggesting these are *trans*-regulatory hotspots.

This study leads us to suggest that the relationship of coding sequence conservation and functional similarity may not be true for *cis*-regulatory sequences. Indeed, recent work as shown this to be the case for enhancers [31]. The problem, however, is that there is not a precise quantitative framework for the interpretation of *cis*-regulatory variation.

## Conclusion

In summary, we have characterized the frequency and diversity of proximal promoter polymorphisms on a genome-wide scale and shown that they do not differ between *D. melanogaster *strains with divergent gene expression. We conclude that sequences elsewhere, such as in *trans*, may cause differential gene expression. Indeed, in the very strains examined in the present study, extensive nonadditivity of gene expression among these strains and their reciprocal F_1 _hybrids indicates that transcription is controlled predominantly by *trans*-regulatory factors [1]. Linkage analysis might allow for mapping of regulatory regions outside the proximal promoter that cause expression variation within the *D. melanogaster *transcriptome.

## Methods

### Microarray data analysis

The data are from the work of Gibson *et al*. [1] with Agilent 60-mer oligonucleotide microarrays, and are available online [32]. *P*-values were calculated from the NLP (Negative Log *P*) values reported by Gibson *et al*. [1], and used to sort the data into two genesets whose expression reproducibly varied at a highly significant level (*P *< 0.01) or not at all (*P *> 0.1) between the two strains. Genes whose significance values fell in between these *P *values were ignored. Genes for which the effect of sex and/or sex by line interaction was significant (*P *< 0.05) were also excluded. Also, gene *CG4109 *was removed from this analysis because it was missing expression and significance values. For each gene in the first geneset, we identified the strain whose Least Squares Mean value was higher and designated it as the strain with greater expression.

From the genesets, we chose autosomal, protein-coding genes whose expression pattern was the same for at least three to five replicate probes, except for gene *CG31908*, which was probed only twice. To avoid ascertainment bias on the basis of known regulatory variation or lack thereof, only those genes whose regulation had not yet been studied were chosen for this study. Of these, 34 genes (ten whose expression is greater in the Russian 2b strain, nine in the Oregon R strain, fifteen whose expression does not vary) were chosen for inclusion. The expected number of genes whose expression varied between the two strains by chance alone was calculated by multiplying the *P *values at each selection criterion by the total number of tests (genes in the microarray) and the number of selection criteria: *e.g*., expected number of genes that vary = (0.01)*(0.95)*(0.95)*(12017)*(3) = 325. The False Discovery Rate (FDR), an indicator of the number of significant features that are truly null [33], was calculated by dividing the expected number by the observed: e.g., FDR = 325.36/483 = 0.6736 = 67.4%.

### Flies

The strains from which the foregoing data were collected, Oregon R and Russian 2b, were obtained from Gregory Gibson at North Carolina State University. Before Gibson *et al*. [1] began their work, these strains were inbred by sib-mating for over a hundred generations and are more isogenic than isofemale lines (G. Gibson, personal communication). Flies were reared on standard cornmeal diet at room temperature without undergoing any experimentally imposed treatments.

### Gene amplification and sequencing

Gene-specific primers for amplification and sequencing were designed with Oligo 4.0 (National Biosciences, Inc.) and gene sequences in FlyBase Release 3.0 [34]. During the course of this study, the entry for *tensin *was annotated and updated. Accordingly, the sequencing and analysis of this gene was modified to accommodate these changes. Primers were synthesized by Integrated DNA Technologies (Iowa City, IA) or at The University of Chicago Oligonucleotide Synthesis Core Facility.

Genomic DNA was extracted *en masse *from 50–100 male or female flies of either strain according to Lerman *et al*. [8] or using a DNeasy Tissue Kit (Qiagen). Between 1–2 kb of the proximal promoter for each gene was amplified with Taq DNA Polymerase (Promega), MasterAmp Extra Long DNA Polymerase Mix (Epicentre Technologies), Pfx (Invitrogen), or Pfu (Stratagene) according to the manufacturers' instructions. Promoter polymorphisms found in more than half (9/15) of the sequences amplified with Taq were verified with a proofreading polymerase. Because these nine sequences amplified with Taq were identical to those amplified with a proofreading polymerase, we assumed the remaining six sequences amplified with Taq also do not contain amplification errors. PCR products were cleaned with QIAquick PCR Purification Kits (Qiagen) or a Sephadex G-50 column and sequenced at The University of Chicago Cancer Research Center DNA Sequencing Facility with the original primer pair used for amplification and two pairs of internal primers. Primer sequences and PCR protocols are available upon request.

### Bioinformatics

Overlapping sequences were compiled and edited using Sequencher 4.1 (Gene Codes, Corp.). The consensus sequences for each gene in both sexes of either strain were aligned to each other and to the Celera strain [35] from FlyBase Release 3.0, used as a reference, with ClustalW on Biology Workbench 3.2 [36] with default alignment parameters. To reduce PCR error and verify the identity of the promoter polymorphisms, we independently amplified and sequenced both sexes. For 25/34 genes (74%), both forward and reverse strands of the promoter in both sexes of both strains were sequenced multiple times, thus obtaining at least 4X coverage. For the remaining 9 genes *(Cng, Scab, eyg, esg, Fkbp13, fkh, pk, toy, CG31908)*, sequence reads covered the promoter region at least twice in each strain, either by covering both strands in one sex or one strand in both sexes. The additional sequencing beyond 2X coverage did not change the base calls as no ambiguous bases were observed. Numbers of promoter polymorphisms (transition SNPs, transversion SNPs, indels) were tabulated and compared between the two genesets with a binomial model (R version 2.0.0; [37]). The two genesets were logit transformed before applying the following Generalized Linear Model:

glm(formula = Y1 ~ indel + transition_SNP + transversion_SNP, family = binomial(link = 'logit'))

where gene expression (the dependent variable X) varies according to the number of each indel, transition SNP or transversion SNP (each as the independent variable Y).

The MatInspector Tool v2.2 was used to search Genomatix Suite [38] for putative transcription factor binding sites created or abolished by the promoter polymorphisms. This database is based upon TRANSFAC and consensus sequences for putative transcription factor binding sites found in the scientific literature (Cartharius *et al*., unpublished). All matrices were searched with default settings; however only those putative transcription factor-binding sites with a core similarity of 0.75 or greater are reported here.

Insertions longer than 12 nt and full-length sequences of the promoters of four genes (*Scab, Cry, Ih, bin*) were BLASTed against a transposable element (TE) database for *Drosophila *extracted from the NCBI nr database (J.-C. Walser, pers. comm.). Only those hits with 100% similarity are included in this study. The direction of insertion or deletion cannot be determined from the data here as there is no outgroup for comparison.

## Authors' contributions

R.P.B. conducted all experimental procedures including mining microarray data and selecting genes of interest, amplifying, sequencing and analyzing the proximal promoters, conducted bioinformatics and statistical analysis, and drafted the manuscript. M.E.F. helped conceive the study and draft the manuscript. Both authors read and approved the manuscript.

## Supplementary Material

Additional File 1 Supplementary Table.doc is a Microsoft Word document that lists and categorizes the sequences of the insertions/deletions between two strains found in the proximal promoter regions of 34 genes. Click here for file
