# Supplementary material for: Reverse transcriptional profiling: non-correspondence of transcript level variation and proximal promoter polymorphism
Source: BMC Genomics. 2005 Aug 17;6:110. doi: 10.1186/1471-2164-6-110 (PMC1192798; doi:10.1186/1471-2164-6-110)
Supplement: Additional File 1 — Supplementary Table.doc is a Microsoft Word document that lists and categorizes the sequences of the insertions/deletions between two strains found in the proximal promoter regions of 34 genes. [file 1471-2164-6-110-S1.doc]

### Supplementary Table

**Comparison of the indels between Oregon R and Russian 2b strains.** Indels are shown with at least 10 bp of flanking sequences to provide a context. Gene = gene name; site = position of 3’ nucleotide flanking the indel, shown in **bold**, from the translational start site [in the case of multiple indels in a gene, as in KP78a, inserted nucleotides between translational start site and upstream indels were counted and deletions ignored]; str = strain, ORR = Oregon R, R2b = Russian 2b; class = based on the categories described in Schaeffer (2002); nr = non-repetitive, hpr = homopolymer repeat, mcs = microsatellite, vntr = variable number of tandem repeats; len=length in nucleotides. An asterix indicates the sequence in the Celera strain.

## Gene site str sequence class len

Fer2 -521 ORR AAATAAATCGGGATATATATTTTAAAA----------**C**ACAATAAAATT* nr 10

R2b AAATGAATTGTGATATATATTTTAAAAGGTATGAAAT**C**ACAATAAAATT

Scab -673 ORR TTAAAGTTTT---------------------------------**A**TTGTTATTA* nr 33

R2b TTAAAGTTTTGGGTAATAGCTATATAAATAGATGTATAATGTT**A**TTGTTATTA

tacc -106 R2b ATCCACAAAAAAAAAAAAAAAAA---------**C**ACTAAAGACCAAACTAA  hpr 6-12

ORR AACCACAAAAAAAAAAAAAAAAAAAAAAAAAA**C**ACTAAAGACCAAACTAA

onecut -1096 R2b ATTTAAAAAAAG----------------------**T**ACTTTCTCGTACTGC nr 22

ORR ATTTAAGCAGTGAACAATTCAGTAGATCCCAGCT**C**ACTTTCTCGTACTGC*

qkr58E-3 -1067 ORR TGTTTGTTTT---**C**TTTGCGAATCAACAAT hpr 3

R2b TGTTTTTTTTTTT**C**TTTGCGAATCAACAAT

esg -1624 ORR TAACTTTGCAAGAAG---**G**CCTATTAAGAG mcs 3

R2b TAACTTTGCAAGAAGAAG**G**CCTATTAAGAG*

KP78a -740 R2b AGTTTTTTTTTT-**G**GCATTCCCTTACAATT* hpr 1

ORR AGTTTTTTTTTTT**G**GCATTCCCTTACAATT

KP78a -1333 R2b AGGCCCTTACACACACACACACACAC----**G**CACACAAAG mcs 4

ORR AGGCCCTTACACACACACACACACACACAC**G**CACACAAAG

KP78a -1484 ORR TGAGAGAGGGA--------**T**ACGCAAAGGA dr 8

R2b TGAGAGAGGGAGAGAGGGA**T**ACGCAAAGGA*

Ih -491 R2b TTGAAAAGGATGTTAA-**A**AAAAAACAGCTT* nr 1

ORR TTGAAAAGGATGTTAAT**A**AAAAAACAGCTT

Ih -298 ORR TCTTGAAGTCACGAGAC-------------**A**ATAATTAGC nr 13

R2b TCTTGAAGTCACGAGACCGCTGAAGTCAAC**A**ATAATTAGC*

Fkbp13 -1314 R2b GAGTCCCAAAAAGGAA------**G**TTGAATTTGGCCGCGAG nr 6

ORR GAGTCCCAAAAAGGAATCGCCT**G**TTGAATTTGGCCGCGAG*

Fkbp13 -324 ORR AAAAATAAAC--**T**AATAAATTAAATTAGAG nr 2

R2b AAAAATAAACTG**T**AATAAATTAAATTAGAG*

Fkbp13 -114 R2b CTACGGCATATTCTTTGG----**A**AACTCCTTTTAGTTAAA* nr 4

ORR CTACTGCATATTCTTTGGTCGC**A**AACTCCTTTTAGTTAAA

fkh -452 ORR ACAACAAAAATCAC-**A**TACAAAAATACAAA hpr 1

R2b ACAACAAAAATCCCC**A**TACAAAAATACAAA*

Mt2 -152 ORR ATATTCTTCTTCTT-----------**A**TACCAATCACACGG* nr 11

R2b ATATTCTTCTTCTTCTGCGCGGACG**A**TACCAATCACATGG

pk -1291 ORR TTCGAATATTATTT---------------------------------**T**ATTAGATTCGAC dr 33

R2b TTCGAATATTATTTAGATTGAATATAATTAGACTGATTAGATTAGAT**T**ATTAGATTCGAC*

stan -292 ORR ATATATTTTTTTTT--**C**AGCAGGCGACAAT hpr 2

R2b ATATATTTTTTTTTTT**C**AGCAGGCGACAAT

stan -61 ORR CACACACACACACACACACA--**T**TCACGGGGA mcs 2

R2b CACACACACACACACACACACA**T**TCACGGGGA*

Cry -1095 R2b ACAATTTGTGGTCATTGGC---**T**TGCTTTTCCTGTTTCTA* dr 3

ORR ACAATTTGTGGTCATTGGCGGC**T**TGCTTTTCCTGTTTCTA

Cry -1044 R2b TTAAGCACA--------------**T**TTAAATCTATATAAAT* nr 14

ORR TTAAGCACAATCTATTACCTACA**T**TTAAATCTATATAAAT

Cry -931 R2b TCAAAAATGG-------------------------------------------**G**GCGTAGCAA* nr 43

ORR TCAAAAATGGCGTGGGCGTGGTAGTTTTGGTCGGTTGTAGGGCGTTTAGAGTG**G**GCTTAGCAA

Cry -801 ORR AATCATAAATCAATCGCCTGC-----**G**TGAAACAAGGAAGCCCCCCAAAA nr 5

R2b AATCATAAATCAATCGCCTGCATTGC**G**TGAAACAAGGAAGCCTCCCAAAA*

Cry -539 ORR TACTTGTGGTCAGCTATAGAATTTCCCCCTTTTTTT--**G**CCGCCTATACC hpr 2

R2b TACTTGTGGTCAGCTATCGAATTTCCCCCTTTTTTTTT**G**CCGCCTATACC*

Cry -499 R2b AACGCCTGGTTACTTTTT----------**A**AGCATTTTGCAATCGGATCTC* hpr 10

ORR AACGCCTGGTTTCTTTTTTTTTTTTTTT**G**AGCATTTTGCAATCGGATCTC

bin -1163 ORR GATCCACATGGCC--------------------**C**ACAGGGCCCGATCTTG nr 20

R2b GATCCACATGGCCATACCCGTACCCGTACCCAT**T**CCCATGCCCGATCTTG

bin -573 R2b CGCATTACATTTTATTTT-----**G**ATACCAACTT hpr 5

ORR CGCATTACATTTTATTTTTTTTT**G**ATACCAACTT

bin -547 ORR ATAGATCTCGATTT-**C**CCTTGAGTTCCGTTTGGGTT hpr 1

R2b ATAGATCTCGATTTT**C**GCTTGAGTTCCGTTTGGGTT*

bin -66 R2b TATTGATAAAAAAAAA-**C**CAAAAATAT hpr 1

ORR TATTGATAAAAAAAAAA**C**CAAAAATAT*
